# Diabetes Distress and Transition Readiness in Youths with Type 1 Diabetes Transitioning from Pediatric to Adult Care

**DOI:** 10.1155/2023/5580180

**Published:** 2023-09-25

**Authors:** Joseph M. W. S. Leung, Naseem Y. Al-Yahyawi, Heywood S. Choi, Laura L. Stewart, Jeffrey N. Bone, Tricia S. Tang, Shazhan Amed

**Affiliations:** ^1^Division of Endocrinology, Department of Medicine, The University of British Columbia, Vancouver, BC, Canada; ^2^Division of Endocrinology and Diabetes, Department of Pediatrics, The University of British Columbia, Vancouver, BC, Canada; ^3^Abbotsford Regional Hospital, Abbotsford, BC, Canada; ^4^Department of Obstetrics and Gynecology, The University of British Columbia, Vancouver, BC, Canada; ^5^BC Children's Hospital Research Institute, Vancouver, BC, Canada

## Abstract

**Background:**

Youths with type 1 diabetes transitioning from pediatric to adult care are known to experience significant glycemic excursions and medical complications. Diabetes distress and transition readiness are two potentially related constructs involved in this transition process, but the relationship between them has not been extensively studied. *Hypothesis*. Lower diabetes distress is associated with increased transition readiness among youths with type 1 diabetes transitioning to adult care.

**Subjects:**

One hundred one adolescents and emerging adults with type 1 diabetes transitioning to adult care complete data in 63 study participants.

**Methods:**

In this cross-sectional study, we collected diabetes distress scale scores (via T1-DDS) and transition readiness scores (via Am I ON TRAC) at the last pediatric diabetes visit. We fitted regression models to estimate the relationship between T1-DDS scores and ON TRAC scores.

**Results:**

The total mean T1-DDS score was associated with ON TRAC knowledge score (*β* = −2.73, 95% CI −4.41,−1.06, *p*=0.002), behavior score (*β* = −2.61, 95% CI −4.39,−0.84, *p*=0.005), and transition readiness indicator (*β* = −0.18, −0.34,−0.01, *p*=0.03). Multiple T1-DDS subscales were associated with ON TRAC knowledge score: powerlessness, management distress, negative social perceptions, eating distress, physician distress, and family/friend distress. Multiple T1-DDS subscales were also associated with ON TRAC behavior score: management distress, negative social perceptions, eating distress, and family/friend distress.

**Conclusions:**

Diabetes distress and transition readiness have an inversely proportional relationship in youths with type 1 diabetes transitioning to adult care. Targeting diabetes distress may also improve transition readiness (and vice versa) in this population.

## 1. Introduction

Adolescents and emerging adults with type 1 diabetes mellitus undergoing the transition to adult diabetes care are known to have worsening glycemic control, which does not stabilize until at least the late twenties [1]. Several well-designed studies have evaluated resource-intensive clinical interventions to improve the transition between pediatric and adult care [2]. However, there are still no widely accepted models of transition that result in lasting and reproducible improvements in glycemic control [3]. One reason for this might be because diabetes distress and transition readiness have not been adequately addressed in this population [4–6]. Despite ongoing calls to routinely assess both of these constructs in clinical practice and research trials, many studies in type 1 diabetes adolescent transition care report diabetes distress only [7], transition readiness only [8], or neither [9].

Diabetes distress refers to the hidden emotional burdens, stresses, and worries that result from managing the chronic self-care demands of diabetes [4]. Emotions related to diabetes distress include frustration, hopelessness, anger, guilt, and fear [10, 11]. Diabetes distress can affect diabetes self-management, access to care, and interpersonal relationships; is positively correlated with HbA1c levels (A1C); and is experienced by up to 67% of adolescents living with type 1 diabetes [10]. Similarly, transition readiness is defined as the adequate acquisition of knowledge and skills to enhance autonomy, personal responsibility, and independence in preparation for the transition to adult-oriented health care [12]. Transition readiness is known to be associated with older age [13, 14], female sex [14], greater self-efficacy in diabetes care [15], greater treatment adherence [15], and lower parental involvement in diabetes care [15].

There is increasing evidence that both diabetes distress and transition readiness play a role in the adolescent transition process, since both appear to be involved in the motivation for self-care, competency in diabetes management, and social support [16, 17]. However, the relationship between these two concepts remains to be clarified. The objective of this study was to determine the relationship between diabetes distress and transition readiness in a cohort of adolescents and emerging adults with type 1 diabetes transitioning from pediatric to adult diabetes health services. Given the areas of overlap, we hypothesized that lower diabetes distress is associated with increased transition readiness.

## 2. Methods

### 2.1. Subjects

This study was approved by the University of British Columbia's Research Ethics Boards and reports the baseline data from a larger study evaluating a type 1 diabetes adolescent transition intervention. After obtaining informed consent, adolescents and emerging adults attending their last pediatric diabetes visit were prospectively recruited from pediatric diabetes clinics at an urban tertiary level children's hospital and a suburban community hospital 75 km away. Patients were included if they had type 1 diabetes for greater than 6 months, had at least one A1C in the last year, and could provide informed consent in English. Patients were excluded if they were pregnant or lactating or if it was known that they would leave the province in the year following their last pediatric diabetes clinic visit (e.g., for school or for work).

### 2.2. Protocol

At the last pediatric diabetes visit, we conducted a clinical chart review to obtain the following clinical data: age at transition, age at diabetes, diagnosis, sex, last three A1Cs, clinic site (tertiary vs. community), insulin regimen (conventional, multiple daily injections, or insulin pump), and comorbid conditions (e.g., thyroid disease, celiac disease, depression, and anxiety). Participants at neither clinic site (tertiary vs. community) received structured care focused on the transition to adult care prior to the last pediatric diabetes visit. Point-of-care A1Cs were obtained in clinic using the Siemens DCA Vantage Analyzer. Using the REDCap platform [18], study participants were emailed a link to online surveys, which included a demographics form, the T1-DDS [19], and the ON TRAC questionnaire [20]. The self-reported demographics form collected data on ethnicity, parental marital status, parental education level, and morbidity. Altogether, the surveys took ∼20–30 min to complete. Participants received a $25 gift card (Canadian dollars) if all surveys were completed.

### 2.3. Survey Instrument for Diabetes Distress

Several scales have been developed to measure diabetes distress, including Diabetes Distress Scale (DDS), Problem Areas in Diabetes (PAID), PAID-Teen (PAID-T), Diabetes Stress Questionnaire for Youths (DSQY), and Issues Coping with Diabetes (ICD), each with different emphases [10]. For instance, DDS focuses on physician, regimen, and interpersonal distress, while PAID highlights treatment, food, and social support problems [11]. The Type 1 Diabetes Distress Scale (T1-DDS) is a more recently established DDS developed for and validated in adults living with type 1 diabetes, but has not been widely used in adolescents and emerging adults [19, 21]. We selected the T1-DDS as our measure because it captures type 1 diabetes-specific dimensions of distress that are relevant for our target population, such as hypoglycemia distress and eating distress [21].

The T1-DDS consists of 28 items on a 6-point Likert scale [19]. Example items include “Feeling that I am not taking as much insulin as I should” and “Feeling frightened that I could have a serious hypoglycemic event when I'm asleep.” The T1-DDS has an internal consistency (Cronbach's alpha) of 0.92 and is scored on seven subscales (internal consistency followed by correlation with total distress (*R*) in parentheses): powerlessness (0.87, 0.89), management distress (0.81, 0.68), hypoglycemia distress (0.75, 0.71), negative social perceptions (0.85, 0.68), eating distress (0.88, 0.80), physician distress (0.77, 0.60), and family/friend distress (0.76, 0.62). T1-DDS scores were reported as a total mean score for the complete T1-DDS (possible score range 1–6), as well as a mean score for each of the seven subscales (possible score range 1–6) [19].

### 2.4. Survey Instrument for Transition Readiness

Several measures of transition readiness have been developed, such as Transition Readiness Assessment Questionnaire (TRAQ) [22], Self-Management and Transition to Adulthood with *R*_*X*_ = Treatment (STAR_*X*_) [23], and Am I ON TRAC (ON TRAC) [20], each with its own theoretical underpinnings. TRAQ is based on the Stages of Change model [24], while STAR_*X*_ is based on the Got Transition model [23] and ON TRAC is based on the ON TRAC framework [25]. We selected the ON TRAC questionnaire to measure transition readiness because its “formative conceptualization” is considered more natural [12]. It was also developed and validated at our institution, which affirms the validity of this measure in our demographic context [20].

The ON TRAC questionnaire (youth version) includes three subscores: knowledge score, behavior score, and transition readiness indicator [20]. The knowledge score is derived from 14 items on a 4-point Likert scale (possible score range 14–56) and included items such as “I can describe my condition to others” and “I know how my health condition affects my physical activities.” The behavior score is derived from nine items on a 5-point Likert scale (possible score range 9–45) and included items such as “I meet with my health care providers on my own” and “I ask health care providers questions about my health at my visits.” The transition readiness indicator is derived from the behavior score as follows: participants received one point if their response was “sometimes” or higher for four of the items and “often” or higher for the remaining five items. Those with a score of eight points or higher were considered “ready for transition” (value = 1) and those with a score of seven points or lower were considered “not ready for transition” (value = 0). The internal consistency (Cronbach's alpha) of the knowledge score is 0.84. The knowledge score is significantly correlated with the behavior score (Pearson's correlation coefficient *r* = 0.53) and the transition readiness indicator (Pearson's correlation coefficient *r* = 0.41) [20].

### 2.5. Data Analysis

#### 2.5.1. Part A: Descriptive Statistics

Summary statistics were generated for each variable, with counts and percentages reported for categorical variables and means and standard deviations reported for continuous variables. We also calculated the proportion of study participants with at least moderate diabetes distress, which is defined as a T1-DDS score of ≥2 [19]. Stata 15.1 (College Station, TX) was used to generate plots and conduct all statistical analyses.

#### 2.5.2. Part B: Main Analyses

We used univariable (unadjusted) and multivariable (adjusted) linear regression to estimate the relationship between the T1-DDS scores (total mean score and subscale scores) and each of ON TRAC knowledge score, behavior score, and transition readiness indicator. For the sake of the statistical analysis, we specified T1-DDS scores as the independent variable and ON TRAC scores as the dependent variable; however, because this was a cross-sectional study, we were aware of the potential bidirectional nature of this relationship (see Section 4.3 in the Section 4).

Potential confounders in the adjusted analyses were selected based on prior knowledge and are as follows: (i) age at transition was considered a possible confounder because it may be associated with transition readiness [20]; (ii) sex was considered a possible confounder because females are known to have higher diabetes distress scores [11, 26]; (iii) average A1C was considered a possible confounder because those with higher A1Cs are known to have higher diabetes distress [27]; and last, (iv) clinic site (tertiary vs. community) was considered a possible confounder because we observed differences between those recruited from the tertiary hospital diabetes clinic and the community hospital diabetes clinic (see Section 3).

We used a standard significance level of *p* < 0.05 in our analyses. However, because we fitted multiple regression models between each combination of T1-DDS subscale and ON TRAC domain, we also display results corresponding to a Bonferroni-corrected significance level of *p* < 0.006. For the Bonferroni correction, we used *α* = 0.05 and *m* = 8 (one for each of the seven T1-DDS subscale plus one for the T1-DDS total mean score), which results in a corrected *p*-value of 0.006.

For the relationship between the T1-DDS total mean score and ON TRAC knowledge score, behavior score, and transition readiness indicator, we explored if there were interactions with (i) age at transition, (ii) sex, (iii) average A1C, and (iv) clinic site. For these analyses, we fitted linear regression models with interaction terms between T1-DDS total mean score and each of (i) age at transition, (ii) sex, (iii) average A1C, and (iv) clinic site.

#### 2.5.3. Part C: Secondary Analyses

We used univariable (unadjusted) and multivariable (adjusted) linear regression to determine if age at transition, sex, and average of last three A1Cs is associated with the T1-DDS total mean score and each T1-DDS subscale, as well as if they are associated with transition readiness (i.e., ON TRAC knowledge score, behavior score, and transition readiness indicator). We conducted these secondary analyses in order to better understand how different clinical characteristics are related to different domains of diabetes distress and transition readiness. Analyses with clinic site as a predictor for T1-DDS and ON TRAC scores will be reported in a future article. As in Part B, we display results based on a standard significance value of *p* < 0.05, as well as the Bonferroni-corrected *p* < 0.006.

#### 2.5.4. Part D: Missing Data

Missing data were addressed using the available case method. To compare those with complete data to those with at least one missing data point, we created a new variable called “missing.” The value of “missing” was “0” if there were no missing data and “1” if there was at least one missing data point. We used logistic regression models to estimate the relationship between each variable and “missing.”

## 3. Results

### 3.1. Part A: Descriptive Statistics

**Figure 1:**
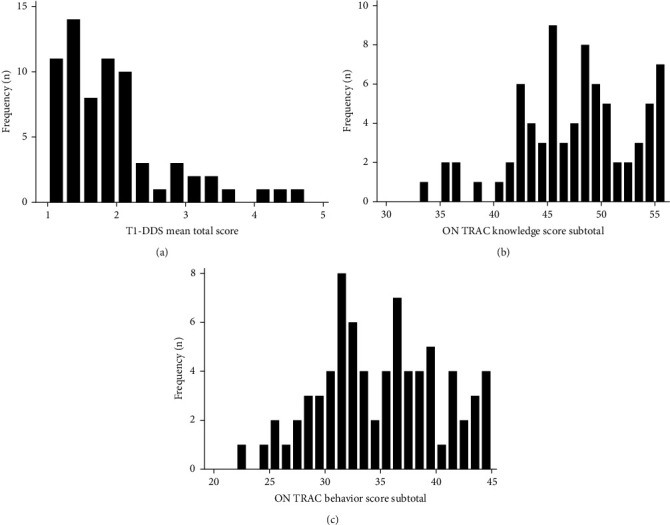
Distribution of (a) T1-DDS, (b) ON TRAC knowledge score, and (c) ON TRAC behavior score.

Between July 2016 and July 2018, we recruited 101 adolescents and emerging adults with type 1 diabetes who were attending their last pediatric diabetes visit.  Table 1 shows the characteristics of all study participants.  Table 1 also subdivides the participants into tertiary versus community site to demonstrate the differences between the two groups.  Figure 1 shows the distributions of the T1-DDS total mean score, ON TRAC knowledge score, and ON TRAC behavior score for all participants. For the ON TRAC transition readiness indicator, 63% were “not ready to transition” and 37% were “ready to transition.” Table 2 displays the proportion of study participants with significant diabetes distress across all T1-DDS subscales.

### 3.2. Part B: Main Analyses

**Figure 2:**
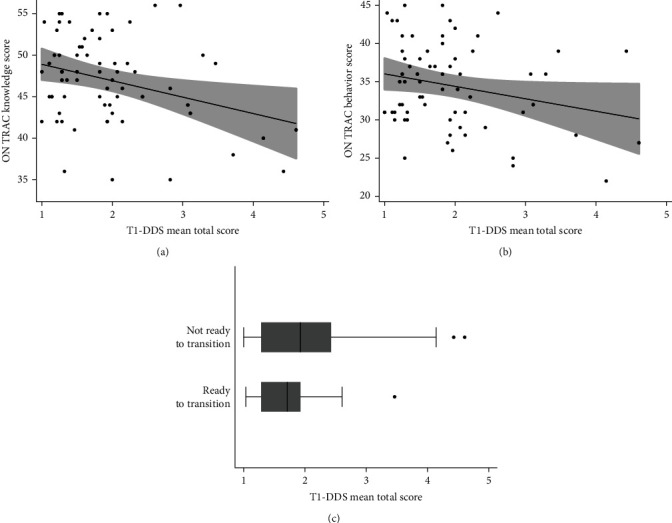
Relationships between diabetes distress and transition readiness: (a) scatter plot of T1-DDS scores and ON TRAC knowledge scores. Gray-shaded area is 95% confidence interval of black trend line; (b) scatter plot of T1-DDS scores and ON TRAC behavior scores. Gray-shaded area is 95% confidence interval of black trend line; (c) box plot of T1-DDS scores and ON TRAC transition readiness indicator.

Beta coefficients (*β*), 95% confidence intervals (95% CI), and *p*-values are reported for the regression models demonstrating the relationship between T1-DDS scores and ON TRAC scores (see Table 3).  Figure 2 displays these relationships graphically. Models are both unadjusted and adjusted for the four potential confounders mentioned above.

In the relationship between T1-DDS total mean score and ON TRAC knowledge score, there were no statistically significant interactions between T1-DDS total mean score and each of: age at transition, average A1C, and clinic site (*p*-values for interaction were all >0.05). However, there was a statistically significant interaction between T1-DDS total mean score and sex (*p*-value for interaction = 0.047). Among females, there was a statistically significant relationship between T1-DDS total mean score and ON TRAC knowledge score (*β* = −3.55, 95% CI −5.32,−1.78–1.06, adjusted *R*^2^ = 0.32, *p* < 0.001). However, among males, there was no statistically significant relationship between T1-DDS total mean score and ON TRAC knowledge score (*β* = 0.82, 95% CI −4.07, 5.71, adjusted *R*^2^ = −0.09, *p*=0.74).

In the relationship between T1-DDS total mean score and ON TRAC behavior score, there were no statistically significant interactions between T1-DDS total mean score and each of: age at transition, sex, average A1C, and clinic site (*p*-values for interaction were all >0.05). In the relationship between T1-DDS total mean score and transition readiness indicator, there were no statistically significant interactions between T1-DDS total mean score and each of: age at transition, sex, average A1C, and clinic site (*p*-values for interaction were all >0.05).

### 3.3. Part C: Secondary Analyses

Beta coefficients (*β*), 95% CI, and *p*-values are reported for the regression models demonstrating the relationship between age at transition, sex, and average A1C and T1-DDS scores (see Table 4). Models are both unadjusted and adjusted for the other two variables plus clinic site (see Table 4).

In the unadjusted analyses, there were no statistically significant relationships between age at transition and ON TRAC knowledge score (*β* = 0.20, 95% CI −0.71, 1.12, *R*^2^ = −0.01, *p*=0.66), behavior score (*β* = −0.29, 95% CI −1.20, 0.62, *R*^2^ = −0.008, *p*=0.52), and transition readiness indicator (*β* = −0.04, 95% CI −0.12, 0.04, *R*^2^ = −0.003, *p*=0.37). There were no statistically significant relationships between sex and ON TRAC knowledge score (*β* = −0.32, 95% CI −2.85, 2.20, *R*^2^ = −0.01, *p*=0.80), behavior score (*β* = −2.21, 95% CI −4.72, 0.31, *R*^2^ = 0.03, *p*=0.08), and transition readiness indicator (*β* = −0.14, 95% CI −0.37, 0.09, *R*^2^ = 0.007, *p*=0.22). There were also no statistically significant relationships between average A1C and ON TRAC knowledge score (*β* = −0.42, 95% CI −1.21, 0.36, *R*^2^ = 0.002, *p*=0.29), behavior score (*β* = −0.56, 95% CI −1.34, 0.22, *R*^2^ = 0.01, *p*=0.16), and transition readiness indicator (*β* = −0.04, 95% CI −0.11, 0.03, *R*^2^ = 0.003, *p*=0.28).

### 3.4. Part D: Missing Data

There were 63 participants with complete data and 38 participants with at least one missing value, including 32 participants with at least one missing T1-DDS data point and 26 participants with at least one missing ON TRAC data point (see Table S1). Twenty-six percent of T1-DDS data were missing, and 25% of ON TRAC data were missing. However, there were no statistically significant differences in any variable (demographic variables, T1-DDS score, and ON TRAC scores) between those with no missing data and those with at least one missing data point.

## 4. Discussion

### 4.1. Summary of Findings

To our knowledge, this is the first paper to specifically examine the relationship between diabetes distress and transition readiness at the time of the last pediatric diabetes visit. We found that all T1-DDS subscales, except for the hypoglycemia distress subscale, were associated with one or more domains of the ON TRAC transition readiness measure. In particular, management distress, negative social perceptions, and eating distress had strong associations with the ON TRAC knowledge score, ON TRAC behavior score, and ON TRAC transition readiness indicator, even in the adjusted models. In our secondary analyses, we showed that female sex was associated with every T1-DDS subscale, while age at transition was not associated with any T1-DDS subscale. Additionally, average A1C was positively correlated with the powerlessness and management distress subscales of the T1-DDS.

### 4.2. Diabetes Distress and Transition Readiness

While both diabetes distress and transition readiness are different constructs, our findings suggest that they are associated with one another. Not surprisingly, there has been an urgent need for routine diabetes distress screening and subsequent management of diabetes distress among adolescents and emerging adults with type 1 diabetes transitioning to adult care [4]. There has also been advocacy (e.g., from the American Academy of Pediatrics [5] and the Got Transition model [28]) to integrate routine assessment of transition readiness in the care of adolescents with chronic health conditions.

Previous studies have identified different factors associated with transition readiness, including increasing age [24], female sex [14], more frequent communication with healthcare providers [17], increased parental involvement [17], and increased engagement of friends in diabetes management [17]. Our findings further clarify how diabetes distress subscales may also be related to transition readiness. For example, we showed that the T1-DDS subscale powerlessness, defined as the broad sense of discouragement about one's diabetes [19], was associated with the ON TRAC knowledge score. We postulate that powerlessness may be counteracted by resilience and self-advocacy, which includes confidence in disclosing one's diagnosis, ability to explain self-care needs, and independent communication with the healthcare team [17, 29]. In fact, these self-advocacy behaviors may even be associated with improved glycemic control [17]. Future type 1 diabetes adolescent transition interventions should consider resilience and self-advocacy training to reduce feelings of powerlessness experienced by this target population.

Management distress is defined as disappointment in one's own diabetes self-care efforts [19]. We found that the T1-DDS subscale management distress was associated with the ON TRAC knowledge score, behavior score, and transition readiness indicator. Other studies have shown that diabetes self-management skills and self-advocacy skills are associated with transition readiness [17, 22]. Our study shows that it is not only self-management skills, but also management distress that is associated with transition readiness. Interestingly, targeting these management skills can improve transition readiness. A longitudinal study found that an 8-month web-based intervention targeting disease management skills improved transition readiness scores (via TRAQ) [30]. While diabetes distress was not measured or specifically targeted in that study, it is possible that interventions addressing management distress would also improve transition readiness.

In our study, negative social perceptions, defined as the concerns related to possible negative judgments because of one's diagnosis and management of type 1 diabetes [19], were also associated with transition readiness. Qualitative studies have identified diabetes stigma and discrimination as impediments to a successful transition and our study quantifies how negative social perceptions are associated with poorer transition readiness [29]. At a life stage when adolescents and emerging adults with type 1 diabetes may face intense peer and social pressure, perceptions of stigma may indicate lack of resilience to address diabetes stigma, which would prevent advancement in transition readiness [31]. Microlevel interventions, such as education to enhance assertiveness and self-efficacy, as well as macrolevel interventions, such as mass media campaigns and reinforcement of antidiscrimination legislation, are tools that may reduce diabetes stigma and diabetes distress, thereby increasing transition readiness [32].

Eating distress, defined as concerns about loss of control with regard to eating, was yet another diabetes distress subscale to be associated with transition readiness in our study. Most transition readiness measures (including the ON TRAC scale that we used) do not include any items related to disordered eating. This is despite the fact that nearly one-quarter of young adults with type 1 diabetes screen positive for disordered eating [33]. Disordered eating is notoriously difficult to identify in individuals with type 1 diabetes and A1C is known to be higher among adolescents with type 1 diabetes who report disordered eating [34]. This highlights the need to screen for and address disordered eating and eating distress among adolescents and emerging adults with type 1 diabetes as these could impact transition readiness and glycemic control.

Physician distress is defined as disappointment with one's healthcare professionals and family/friend distress is defined as loved ones putting too much emphasis on diabetes. Both of these diabetes distress subscales were associated with transition readiness in our study. Because the development of transition readiness in young adults with type 1 diabetes is impacted by diabetes-specific relationship processes, such as provider communication, parental knowledge, and friend helpfulness [17], it is not surprising that diabetes distress in these relationship domains can impact transition readiness. For example, communication style between patients and their providers is one factor that can influence adherence to one's diabetes regimen [17]. As another example, family and friends who may not understand the difficulties in living with diabetes may be viewed as “diabetes police,” which can increase diabetes distress [35]. These findings lead us to believe that the development of individualized and intentional avenues of positive communication with one's support network can improve diabetes distress and transition readiness, which may also translate into improved healthcare outcomes, such as glycemic control [14, 27].

### 4.3. Potential Bidirectionality of Relationship

Throughout the analysis of this study, we considered diabetes distress as the independent variable and transition readiness as the dependent variable. However, because this was a cross-sectional study, transition readiness may have also impacted diabetes distress as well. For instance, in a cross-sectional survey study among young adults with type 1 diabetes who had already made the transition to adult care, those who felt prepared for the transition had less diabetes distress [36]. Another cross-sectional study in a similar population demonstrated that emerging adults who had a poor experience during their transition to adult care had higher diabetes distress [37]. Additionally, we measured diabetes distress at the time of transition (i.e., the last pediatric diabetes visit), which may be a time of high distress and found that 63% of our sample had higher diabetes distress, nearly double the proportion compared to other studies [37]. Put together, the transition period and by extension, transition readiness during this time, may impact diabetes distress in adolescents and emerging adults.

### 4.4. Demographic Variables Associated with Diabetes Distress and Transition Readiness

Consistent with studies in children [38], adolescents [35], and adults [19], our study showed that female youths have higher levels of diabetes distress compared to male youths for every subscale of the T1-DDS at the time of transition to adult care. Another study used the DSQY and showed that adolescent females with type 1 diabetes had higher diabetes distress scores compared with males for five of eight subscales, including distress-worry, adverse interpersonal effects, hyperglycemia, diet, and hypoglycemia [27]. Multiple reasons for higher diabetes distress in females have been previously described, including decreased resilience, increased disordered eating behaviors, increased depressive symptoms, increased pressure for social acceptance, and increased issues with body image [38]. Ongoing efforts are needed to explore how diabetes distress among female youths with type 1 diabetes can be mitigated.

The relationship between higher diabetes distress and higher A1C has been previously observed using the DDS [39], PAID-T [40], and DSQY [27]. Using the T1-DDS, we were able to demonstrate that this A1C-associated diabetes distress was driven mainly by powerlessness and management distress. There has been suggestion that coping strategies such as problem solving and positive thinking can reduce both these types of diabetes distress [41].

As for transition readiness, we did not find any statistically significant relationships between age at transition, sex, or average A1C and any of the ON TRAC transition readiness domains. Wood et al. [24] has previously shown that older age and female sex are associated with transition readiness. We suspect that our sample size was too small to detect these relationships. There have been incongruent reports about the relationship between A1C and transition readiness [14, 17, 42]. We believe that further study is needed to clarify this relationship. Additionally, using a transition outcomes instrument such as the Healthcare Transition Outcomes Inventory (HCTOI) may provide more meaningful data about the success of a youth's transition process and experience than just A1C alone [43].

## 5. Conclusions

We found that higher diabetes distress was associated with poorer transition readiness in a cohort of adolescents and emerging adults with type 1 diabetes at their last pediatric diabetes visit prior to transition to adult care. The consideration of diabetes distress in youths living with type 1 diabetes transitioning to adult care may be a missing link in the development of effective clinical interventions designed to improve transition readiness. These findings amplify the importance of routinely assessing both diabetes distress and transition readiness in clinical practice and should motivate research teams to include both constructs in the design and evaluation of future type 1 diabetes adolescent transition care interventions.

## Figures and Tables

**Table 1:** Characteristics of study participants.

Demographic variable	All participants		Tertiary site		Community site	
	*n*	Value	*n*	Value	*n*	Value
Age at transition, years, mean (SD)	101	19.1 (1.4)	68	18.4 (0.6)	33	20.5 (1.6)
Age at diagnosis, years, mean (SD)	101	9.4 (4.3)	68	8.6 (4.4)	33	10.9 (3.9)
Female sex (%)	101	50 (50)	68	32 (47)	33	18 (55)
Average HbA1c						
% (SD)	101	8.5 (1.5)	68	8.2 (1.5)	33	9.1 (1.5)
mmol/mol (SD)		69 (17)		66 (16)		76 (16)
Clinic						
Academic (%)	101	68 (67)	68	68 (100)	33	0 (0)
Community (%)		33 (33)		0 (0)		33 (100)
Insulin regimen						
Conventional (%)	101^a^	17 (17)	68^a^	14 (21)	33	3 (9)
MDI (%)		33 (33)		23 (34)		10 (30)
CSII (%)		50 (50)		30 (44)		20 (61)
Any medical comorbidity	101	19 (19)	68	14 (21)	33	5 (15)
Any psychiatric comorbidity	101	10 (10)	68	4 (6)	33	6 (18)
Any comorbidity	101	25 (25)	68	17 (25)	33	8 (24)
Ethnicity						
Caucasian (%)	82	60 (73)	54	41 (76)	28	19 (68)
Non-Caucasian (%)		22 (27)		13 (24)		9 (32)
Mother's marital status						
Married (%)	80	58 (73)	52	39 (75)	28	19 (68)
Not married (%)		22 (28)		13 (25)		9 (32)
Mother's education						
Any postsecondary (%)	80	49 (61)	52	30 (58)	28	19 (68)
High school or less (%)		31 (39)		22 (42)		9 (32)
Father's marital status						
Married (%)	77	61 (79)	50	40 (80)	27	21 (78)
Not married (%)		16 (21)		10 (20)		6 (22)
Father's education						
Any postsecondary (%)	77	42 (55)	51	29 (57)	26	13 (50)
High school or less (%)		35 (45)		22 (43)		13 (50)
Number of hospitalizations in last year, mean (SD)	79	0.13 (0.54)	51	0.02 (0.14)	28	0.32 (0.86)
Number of ER visits in last year, mean (SD)	79	0.42 (0.90)	51	0.24 (0.59)	28	0.75 (1.24)
Days missed school in last month, mean (SD)	79	0.43 (1.05)	51	0.45 (0.94)	28	0.39 (1.23)
Days too ill for activities in last month, mean (SD)	79	0.82 (1.82)	51	0.82 (1.65)	28	0.82 (2.13)
Days needing caregiver in last month, mean (SD)	78	0.19 (0.70)	50	0.24 (0.77)	28	0.11 (0.57)

T1-DDS scores	*n*	Value	*n*	Value	*n*	Value

Total mean score, mean (SD)	69	1.9 (0.8)	44	1.8 (0.8)	25	2.1 (0.9)
Powerlessness subscale score, mean (SD)	73	2.4 (1.1)	46	2.3 (1.1)	27	2.5 (1.0)
Management distress subscale score, mean (SD)	76	2.2 (1.3)	48	2.0 (1.2)	28	2.5 (1.2)
Hypoglycemia distress subscale score, mean (SD)	74	1.8 (1.1)	46	1.7 (1.1)	28	2.0 (1.1)
Negative social perceptions subscale score, mean (SD)	75	1.9 (1.1)	48	1.8 (1.1)	27	2.0 (1.0)
Eating distress subscale score, mean (SD)	76	2.1 (1.1)	48	2.0 (1.1)	28	2.3 (1.0)
Physician distress subscale score, mean (SD)	76	1.3 (0.8)	48	1.4 (0.9)	28	1.3 (0.7)
Family/friend distress subscale score, mean (SD)	73	2.0 (1.2)	47	1.9 (1.1)	26	2.2 (1.2)

ON TRAC Transition Readiness Questionnaire	*n*	Value	*n*	Value	*n*	Value

Knowledge score, mean (SD)	76	47.0 (5.4)	49	46.4 (5.6)	27	48.1 (5.0)
Behavior score, mean (SD)	75	34.5 (5.4)	48	34.4 (5.7)	27	34.8 (5.0)
Transition readiness indicator						
No (%)	75	47 (63)	48	30 (63)	27	17 (63)
Yes (%)		28 (37)		18 (38)		10 (37)

*Note*. ^a^One participant was not on insulin at the time of transition.

**Table 2:** Proportion of study participants who met the threshold for diabetes distress.

T1-DDS scores	All participants		Tertiary site		Community site	
	*n*	Proportion	*n*	Proportion	*n*	Proportion
Total mean score, mean (SD)	69	36%	44	32%	25	44%
Powerlessness subscale score, mean (SD)	73	63%	46	59%	27	70%
Management distress subscale score, mean (SD)	76	47%	48	40%	28	61%
Hypoglycemia distress subscale score, mean (SD)	74	32%	46	28%	28	39%
Negative social perceptions subscale score, mean (SD)	75	33%	48	27%	27	44%
Eating distress subscale score, mean (SD)	76	47%	48	42%	28	57%
Physician distress subscale score, mean (SD)	76	13%	48	15%	28	11%
Family/friend distress subscale score, mean (SD)	73	37%	47	36%	26	38%

*Note*. Participants with T1-DDS scores of ≥2 are considered to have significant diabetes distress [19].

**Table 3:** Relationship between T1-DDS scores and ON TRAC scores.

T1-DDS subscale			ON TRAC (unadjusted models)			ON TRAC (adjusted models)		
			KS	BS	TRI	KS	BS	TRI
	Total mean score	*β*	*−1.98*	*−1.63*	−0.11	−2.73	−2.61	*−0.18*
		95% CI	*−3.47, −0.48*	*−3.25, −0.01*	−0.25, 0.03	−4.41, −1.06	−4.39, −0.84	*−0.34, −0.01*
		Adj *R*^2^	*0.08*	*0.04*	0.02	0.13	0.13	*0.04*
		*p*-value	*0.01*	*0.049*	0.12	0.002	0.005	*0.03*

1	Powerlessness	*β*	*−1.35*	−0.74	−0.05	*−1.57*	−1.05	−0.07
		95% CI	*−2.46, −0.24*	−1.96, 0.48	−0.16, 0.05	*−2.81, −0.33*	−2.39, 0.28	−0.19, 0.05
		Adj *R*^2^	*0.06*	0.01	−0.00	*0.07*	0.06	−0.005
		*p*-value	*0.02*	0.23	0.32	*0.01*	0.12	0.25

2	Management distress	*β*	−1.41	−1.61	*−0.10*	−1.82	−2.23	*−0.13*
		95% CI	−2.36, −0.45	−2.56, −0.67	*−0.19, −0.01*	−2.92, −0.72	−3.26, −1.20	*−0.23, −0.03*
		Adj *R*^2^	0.09	0.13	*0.05*	0.12	0.23	*0.08*
		*p*-value	0.004	0.001	*0.03*	0.002	<0.001	*0.01*

3	Hypoglycemia distress	*β*	−0.89	−0.52	−0.02	−1.05	−0.85	−0.05
		95% CI	−2.06, 0.28	−1.71, 0.66	−0.13, 0.08	−2.27, 0.17	−2.06, 0.35	−0.16, 0.07
		Adj *R*^2^	0.02	−0.003	−0.01	0.02	0.04	−0.009
		*p*-value	0.13	0.38	0.65	0.09	0.16	0.42

4	Negative social perceptions	*β*	*−1.33*	−1.68	−0.08	*−1.50*	−2.11	*−0.11*
		95% CI	*−2.47, −0.19*	−2.80, −0.56	−0.19, 0.02	*−2.69, −0.32*	−3.22, −1.01	*−0.22, −0.00*
		Adj *R*^2^	*0.06*	0.10	0.02	*0.07*	0.20	*0.05*
		*p*-value	*0.02*	0.004	0.12	*0.01*	<0.001	*0.048*

5	Eating distress	*β*	−1.90	*−1.33*	−0.08	−2.37	−1.96	*−0.12*
		95% CI	−3.01, −0.79	*−2.48, −0.17*	−0.19, 0.03	−3.57, −1.17	−3.18, −0.74	*−0.23, −0.00*
		Adj *R*^2^	0.13	*0.05*	0.02	0.17	0.15	*0.05*
		*p*-value	0.001	*0.03*	0.13	<0.001	0.002	*0.046*

6	Physician distress	*β*	−2.33	−0.48	−0.06	−2.55	−0.94	−0.10
		95% CI	−3.74, −0.91	−2.00, 1.03	−0.19, 0.08	−4.05, −1.05	−2.51, 0.64	−0.24, 0.05
		Adj *R*^2^	0.12	−0.01	−0.004	0.13	0.04	0.01
		*p*-value	0.002	0.53	0.41	0.001	0.24	0.19

7	Family/friend distress	*β*	−1.52	−0.89	−0.07	−1.87	*−1.32*	−0.11
		95% CI	−2.57, −0.47	−1.99, 0.21	−0.17, 0.02	−3.01, −0.73	*−2.51, −0.14*	−0.21, 0.00
		Adj *R*^2^	0.09	0.02	0.02	0.12	*0.09*	0.03
		*p*-value	0.005	0.11	0.14	0.002	*0.03*	0.054

*Note*. Univariable models are unadjusted. Multivariable models are adjusted for age at transition, sex, average A1C, and clinic site. KS, knowledge score; BS, behavior score; TRI, transition readiness indicator; 95% CI, 95% confidence interval; Adj *R*^2^, adjusted *R*^2^. Italicized values indicate *p* < 0.05. Underlined values indicate *p* < 0.006 (Bonferroni-corrected *p*-value in light of multiple comparisons).

**Table 4:** Relationship between demographic variables and T1-DDS scores.

T1-DDS Subscale			Variables (unadjusted models)			Variables (adjusted models)		
			Age	Sex	Avg A1C	Age	Sex	Avg A1C
	Total mean score	*β*	0.08	−0.69	*0.14*	0.02	−0.63	0.10
		95% CI	−0.07, 0.24	−1.06, −0.33	*0.02, 0.26*	−0.16, 0.21	−0.99, −0.27	−0.01, 0.21
		Adj *R*^2^	0.003	0.17	*0.07*	0.19	0.19	0.19
		*p*-value	0.27	<0.001	*0.02*	0.81	0.001	0.08

1	Powerlessness	*β*	0.10	−0.76	*0.21*	0.07	−0.68	*0.18*
		95% CI	−0.08, 0.28	−1.23, −0.28	*0.06, 0.36*	−0.15, 0.30	−1.15, −0.20	*0.03, 0.33*
		Adj *R*^2^	0.003	0.11	*0.09*	0.15	0.15	*0.15*
		*p*-value	0.27	0.002	*0.007*	0.51	0.0056	*0.02*

2	Management distress	*β*	0.16	−0.93	0.31	0.07	−0.80	0.25
		95% CI	−0.04, 0.37	−1.47, −0.39	0.14, 0.48	−0.17, 0.32	−1.31, −0.28	0.08, 0.42
		Adj *R*^2^	0.02	0.13	0.14	0.22	0.22	0.22
		*p*-value	0.11	0.001	0.001	0.56	0.003	0.004

3	Hypoglycemia distress	*β*	0.03	*−0.58*	0.05	−0.03	*−0.55*	0.02
		95% CI	−0.15, 0.21	*−1.07, −0.09*	−0.11, 0.21	−0.27, 0.20	*−1.06, −0.04*	−0.14, 0.18
		Adj *R*^2^	−0.01	*0.06*	−0.008	0.02	*0.02*	0.02
		*p*-value	0.78	*0.02*	0.53	0.78	*0.03*	0.83

4	Negative social perceptions	*β*	0.05	*−0.63*	0.08	0.01	*−0.60*	0.04
		95% CI	−0.14, 0.23	*−1.12, −0.15*	−0.08, 0.24	−0.23, 0.25	*−1.10, −0.10*	−0.12, 0.21
		Adj *R*^2^	−0.01	*0.07*	−0.0004	0.04	*0.04*	0.04
		*p*-value	0.61	*0.01*	0.33	0.94	*0.02*	0.59

5	Eating distress	*β*	0.10	−0.86	0.14	0.06	−0.81	0.09
		95% CI	−0.08, 0.27	−1.32, −0.41	−0.01, 0.30	−0.16, 0.28	−1.27, −0.35	−0.06, 0.24
		Adj *R*^2^	0.002	0.15	0.03	0.15	0.15	0.15
		*p*-value	0.28	<0.001	0.07	0.61	0.001	0.22

6	Physician distress	*β*	−0.07	*−0.51*	0.07	−0.09	*−0.49*	0.06
		95% CI	−0.21, 0.07	*−0.88, −0.14*	−0.05, 0.19	−0.27, 0.09	*−0.87, −0.11*	−0.06, 0.19
		Adj *R*^2^	0.0001	*0.08*	0.004	0.07	*0.07*	0.07
		*p*-value	0.32	*0.008*	0.26	0.33	*0.01*	0.30

7	Family/friend distress	*β*	0.11	−0.88	*0.17*	0.11	−0.83	0.13
		95% CI	−0.10, 0.33	−1.40, −0.36	*0.00, 0.34*	−0.17, 0.38	−1.36, −0.31	−0.04, 0.29
		Adj *R*^2^	0.001	0.13	*0.04*	0.13	0.13	0.13
		*p*-value	0.30	0.001	*0.04*	0.44	0.002	0.13

*Note*. Univariable models are unadjusted. Multivariable models are adjusted for age at transition, sex, average A1C, and clinic site. Avg A1C, average A1C; 95% CI, 95% confidence interval; Adj *R*^2^, adjusted *R*^2^. Italicized values indicate *p* < 0.05. Underlined values indicate *p* < 0.006 (Bonferroni-corrected *p*-value in light of multiple comparisons).

## Data Availability

The data used to support the findings of this study have been deposited in the Dryad repository as “Type 1 diabetes adolescent transition study” (https://doi.org/10.5061/dryad.tdz08kpzh).

## Additional Points

*Strengths and Limitations*. One strength of this study is the inclusion of adolescents and emerging adults from both tertiary and community pediatric diabetes centers, increasing the generalizability of study findings to diverse care settings. However, our study was conducted only in the Canadian province of British Columbia, limiting the generalizability of our study findings in other jurisdictions. GET-IT-T1D is an ongoing intervention trial that measures both diabetes distress (via T1-DDS) and transition readiness (via ON TRAC) in adolescents living with type 1 diabetes in Montreal, Canada [44]. Results from that trial will help validate the findings from this study. Another strength of our study is the use of the T1-DDS, which includes more type 1 diabetes-specific domains [10].

Limitations of our study include the self-reported nature of these measures, cross-sectional study design, and potentially biased sample, since we did not track youths who were approached by decline to participate. We recruited participants at the last pediatric diabetes visit, which could be a period of high distress, resulting in an overestimation of our diabetes distress scores; however, this may also be a true reflection of diabetes distress in this population undergoing such a dramatic life change. Our effect sizes were also small, which may be related to the relatively small sample size. However, we found that multiple subscales of the T1-DDS were associated with the ON TRAC knowledge score and behavior score.

Last, 38% of participants had at least one missing data value, although those with at least one missing value did not differ from those with complete data for any of the demographic characteristics. In light of this, we did not pursue further methods to address missing data. Nevertheless, because around one-quarter of participants were missing both T1-DDS and ON TRAC data, the results of this study should be considered with caution. Future studies evaluating both diabetes distress and transition readiness will help to confirm the findings from this study [44].

## Supplementary Materials

Supplementary Materials Table S1: Characteristics of study participants with complete data versus at least one missing data point.
