# Supplementary material for: Diabetes Distress and Transition Readiness in Youths with Type 1 Diabetes Transitioning from Pediatric to Adult Care
Source: Pediatr Diabetes. 2023 Sep 25;2023:5580180. doi: 10.1155/2023/5580180 (PMC12016687; doi:10.1155/2023/5580180)
Supplement: Supplementary Materials — Table S1: Characteristics of study participants with complete data versus at least one missing data point. [file 5580180.f1.docx]

**SUPPLEMENTARY TABLES**

**Supplementary Table 1. Characteristics of study participants with complete data vs. at least one missing data point.** *Note: One participant was not on insulin at the time of transition.

|  | **Missing = 0** | | **Missing = 1** | |
| --- | --- | --- | --- | --- |
| **Demographic Variable** | **n** | **Value** | **n** | **Value** |
| Age at transition, years, mean (SD) | 63 | 19.0 (1.3) | 38 | 19.2 (1.6) |
| Age at diagnosis, years, mean (SD) | 63 | 9.6 (4.5) | 38 | 9.0 (4.1) |
| Female sex (%) | 63 | 36 (57) | 38 | 14 (37)  24 (63) |
| Average HbA1c % (SD)  mmol/mol (SD) | 63 | 8.4 (1.6)  69 (17) | 38 | 8.6 (1.5)  70 (16) |
| Clinic Academic (%)  Community (%) | 63 | 42 (67)  21 (33) | 38 | 26 (68)  12 (32) |
| Insulin Regimen Conventional (%)  MDI (%)  CSII (%) | 63* | 8 (13)  23 (37)  31 (49) | 38 | 9 (24)  10 (26)  19 (50) |
| Any Medical Comorbidity | 63 | 12 (19) | 38 | 7 (18) |
| Any Psychiatric Comorbidity | 63 | 6 (10) | 38 | 4 (11) |
| Any Comorbidity | 63 | 17 (27) | 38 | 8 (21) |
| Ethnicity Caucasian (%)  Non-Caucasian (%) | 63 | 45 (71)  18 (29) | 19 | 15 (79)  4 (21) |
| Mother’s Marital Status Married (%)  Not married (%) | 63 | 47 (75)  16 (25) | 17 | 11 (65)  6 (35) |
| Mother’s Education Any post-secondary (%)  High school or less (%) | 63 | 38 (60)  25 (40) | 17 | 11 (65)  6 (35) |
| Father’s Marital Status Married (%)  Not married (%) | 63 | 50 (79)  13 (21) | 14 | 11 (79)  3 (21) |
| Father’s Education Any post-secondary (%)  High school or less (%) | 63 | 34 (54)  29 (46) | 14 | 8 (57)  6 (43) |
| Number of hospitalizations in last year, mean (SD) | 63 | 0.16 (0.60) | 16 | 0 (0) |
| Number of ER visits in last year, mean (SD) | 63 | 0.43 (0.96) | 16 | 0.38 (0.62) |
| Days missed school in last month, mean (SD) | 63 | 0.44 (1.09) | 16 | 0.38 (0.89) |
| Days too ill for activities in last month, mean (SD) | 63 | 0.71 (1.83) | 16 | 1.25 (1.77) |
| Days needing caregiver in last month, mean (SD) | 63 | 0.16 (0.60) | 15 | 0.33 (1.05) |
| **T1-DDS Scores** | **n** | **Value** | **n** | **Value** |
| Total Mean Score, mean (SD) | 63 | 1.9 (0.9) | 6 | 1.7 (0.4) |
| Powerlessness Subscale Score, mean (SD) | 63 | 2.4 (1.1) | 10 | 2.3 (0.8) |
| Management Distress Subscale Score, mean (SD) | 63 | 2.2 (1.2) | 13 | 2.4 (1.5) |
| Hypoglycemia Distress Subscale Score, mean (SD) | 63 | 1.8 (1.0) | 11 | 1.9 (1.4) |
| Negative Social Perceptions Subscale Score, mean (SD) | 63 | 1.8 (1.1) | 12 | 2.3 (1.1) |
| Eating Distress Subscale Score, mean (SD) | 63 | 2.1 (1.1) | 13 | 2.2 (1.1) |
| Physician Distress Subscale Score, mean (SD) | 63 | 1.3 (0.7) | 13 | 1.5 (1.3) |
| Family/Friend Distress Subscale Score, mean (SD) | 63 | 2.0 (1.2) | 10 | 2.2 (0.8) |
| **ON TRAC Transition Readiness Questionnaire** | **n** | **Value** | **N** | **Value** |
| Knowledge Score, mean (SD) | 63 | 47.2 (5.2) | 13 | 46.1 (6.6) |
| Behavior Score, mean (SD) | 63 | 34.7 (5.7) | 12 | 33.8 (3.7) |
| Transition Readiness Indicator No (%)  Yes (%) | 63 | 39 (62)  24 (38) | 12 | 8 (67)  4 (33) |
